# Functional proteomics identify mannitol metabolism in serum resistance and therapeutic implications in *Vibrio alginolyticus*

**DOI:** 10.3389/fimmu.2022.1010526

**Published:** 2022-10-31

**Authors:** Tian-shun Kou, Jia-han Wu, Xuan-wei Chen, Bo Peng

**Affiliations:** ^1^ State Key Laboratory of Biocontrol, Guangdong Key Laboratory of Pharmaceutical Functional Genes, School of Life Sciences, Southern Marine Science and Engineering Guangdong Laboratory (Zhuhai), Sun Yat-sen University, Higher Education Mega Center, Guangzhou, China; ^2^ Laboratory for Marine Biology and Biotechnology, Qingdao National Laboratory for Marine Science and Technology, Qingdao, China

**Keywords:** mannitol, *Vibrios*, serum, proteomics, glycolysis, pyruvate cycle

## Abstract

Serum resistance is recognized as one of the most important pathogenic traits of bacterial pathogens, and no control measure is available. Based on our previous discovery that pathogenic *Escherichia coli* represses glycine, serine, and threonine metabolism to confer serum resistance and that the reactivation of this pathway by exogenous glycine could restore serum sensitivity, we further investigate the mechanism underlying the action of glycine in *Vibrio alginolyticus*. Thus, *V. alginolyticus* is treated with glycine, and the proteomic change is profiled with tandem mass tag-based quantitative proteomics. Compared to the control group, glycine treatment influences the expression of a total of 291 proteins. Among them, a trap-type mannitol/chloroaromatic compound transport system with periplasmic component, encoded by N646_0992, is the most significantly increased protein. In combination with the pathway enrichment analysis showing the altered fructose and mannitol metabolism, mannitol has emerged as a possible metabolite in enhancing the serum killing activity. To demonstrate this, exogenous mannitol reduces bacterial viability. This synergistic effect is further confirmed in a *V. alginolyticus*–*Danio rerio* infection model. Furthermore, the mechanism underlying mannitol-enabled serum killing is dependent on glycolysis and the pyruvate cycle that increases the deposition of complement components C3b and C5b-9 on the bacterial surface, whereas inhibiting glycolysis or the pyruvate cycle significantly weakened the synergistic effects and complement deposition. These data together suggest that mannitol is a potent metabolite in reversing the serum resistance of *V. alginolyticus* and has promising use in aquaculture.

## Introduction

The complement system plays essential roles in innate immunity, and it consists of more than 30 proteins. Three pathways are known to activate the complement system to clear bacterial pathogens, namely: classical pathway, alternative pathway, and lectin pathway (1–3). By these pathways, the complement system not only functions by directly killing the bacteria *via* forming a membrane attack complex at the bacterial surface but also opsonizes bacteria for phagocytosis, wherein antigens are presented to B cells for antibody production (4–6). Thus, the complement system plays critical roles in bridging the innate immune system and the adaptive immune system during a bacterial infection.

Accumulating evidence demonstrate that bacterial pathogens have evolved different mechanisms to evade complement-mediated killing (7, 8). Three successful strategies have been recognized. Bacteria produce proteins like complement-regulator-acquiring protein and M protein family to recruit complement factors like factor H, factor H-like protein-1, and C4-binding protein so that the complement system is disabled (9, 10). Bacteria, especially *Staphylococcal*, express proteins that directly interact with the hub component of the complement system, C3, C5, C3 convertase, or C5 convertase, to inhibit their functions (11, 12) Staphylokinase, *Pseudomonas* elastase, and 56-kDa protease are known bacterial proteases to degrade C3, C3b, C5a, and IgG (13–15). Although the knowledge on serum resistance is expanding, control measures are still lacking due to the varied mechanisms that are hard to be specifically targeted.

Recently, we proposed that metabolic state determines serum resistance (16–19). By comparing serum-resistant and serum-sensitive bacteria, we find that glycine, serine, and threonine metabolism is repressed in serum-resistant bacteria. Glycine is one of the most decreased metabolites in serum-resistant bacteria. Thus, we supplement serum-resistant bacteria with exogenous glycine that reverses serum resistance and increases serum killing efficacy. This synergistic effect is confirmed both *in vivo* and *in vitro*. The underlying mechanism is that glycine depletes intracellular ATP through the inhibition of ATP synthase and downregulates purine metabolism. The reduced ATP, in turn, decreases cAMP/CRP that enhances complement-binding protein expression. However, whether other mechanisms are present in a different species of *E. coli* is unknown, and the identification of the working mechanism would find a new metabolite in reversing serum resistance in Vibrios.

## Results

### Glycine enhances fish serum to kill *Vibrios*

**Figure 1:**
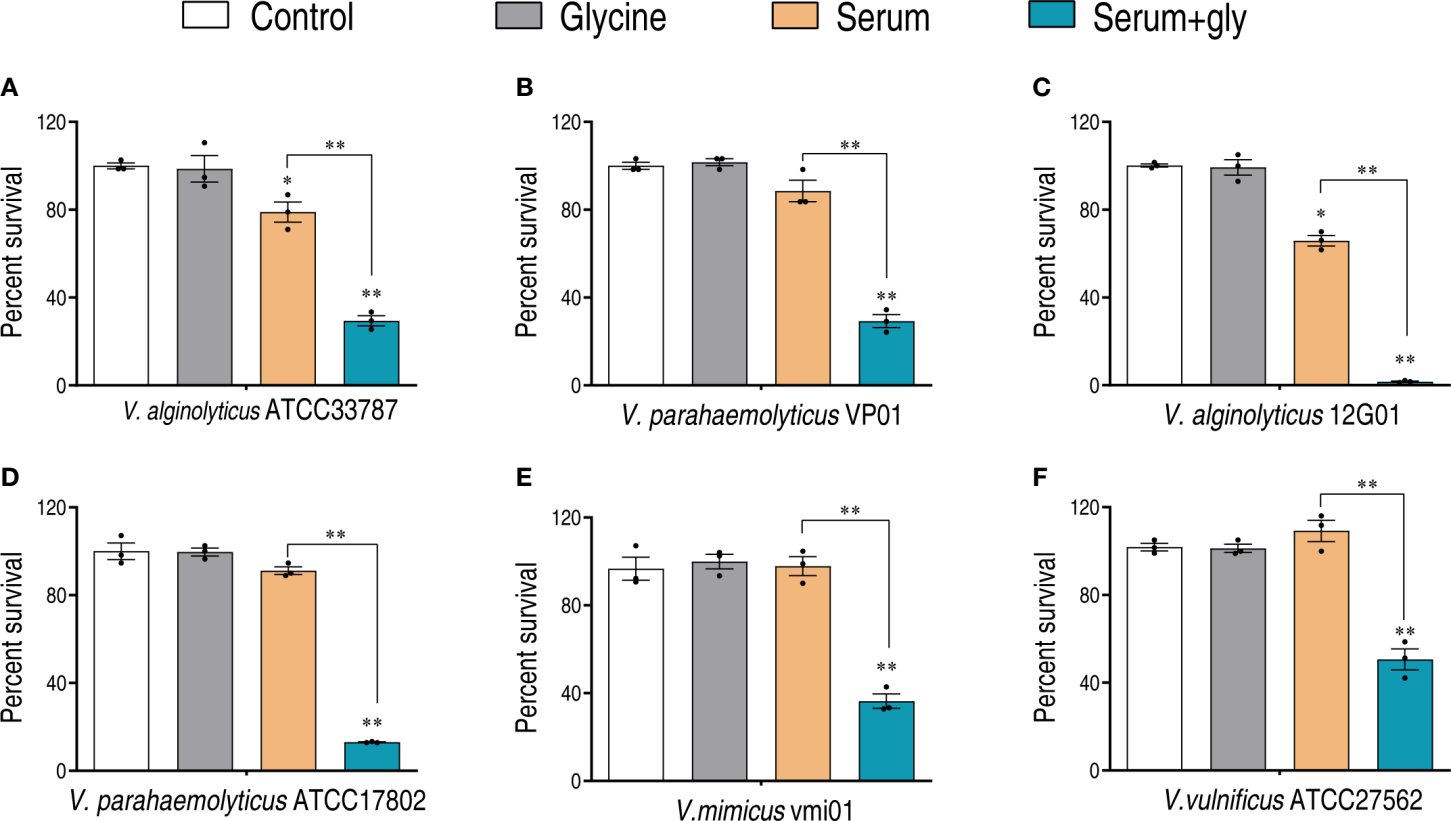
Glycine enhances serum killing to *Vibrio*. Percent survival of *V. alginolyticus* ATCC33787 **(A)**, *V. parahaemolyticus* VP01 **(B)**, *V. alginolyticus* 12G01 **(C)**, *V. parahaemolyticus* ATCC17802 **(D)**, *Vibrio mimicus* vmi01 **(E)**, and *V. vulnificu* ATCC27562 **(F)** in the presence of 100 mM glycine or/and 100 μl serum. The results are displayed as means ± SEM, and significant differences are identified (*p < 0.05; **p < 0.01) as determined by Student’s *t*-test. At least three biological repeats were carried out.

The efficacy of glycine on potentiating serum to kill *Vibrio* pathogens was investigated with our previously established conditions that pathogens were incubated with serum in the presence or absence of glycine (18). Clinic isolates including *V. alginolyticus* (ATCC33787 and 12G01), *V. parahaemolyticus* (ATCC17802 and VP01), *V. mimicus* vmi01, and *V. vulnificus* ATCC27562 were investigated. As shown in **Figure 1**, glycine alone had limited effects on the survival of *Vibrios*, while serum killed a minor portion of the pathogens. The presence of glycine greatly increased the susceptibility of pathogens to serum killing. The viability of the pathogens decreased 2.2–40.1 folds in the presence of glycine as compared to glycine only, whereas *V. alginolyticus* 12G01 was the most susceptible strain that was used for further functional study.

### Proteomic analysis of glycine-treated *V. alginolyticus* 12G01

**Figure 2:**
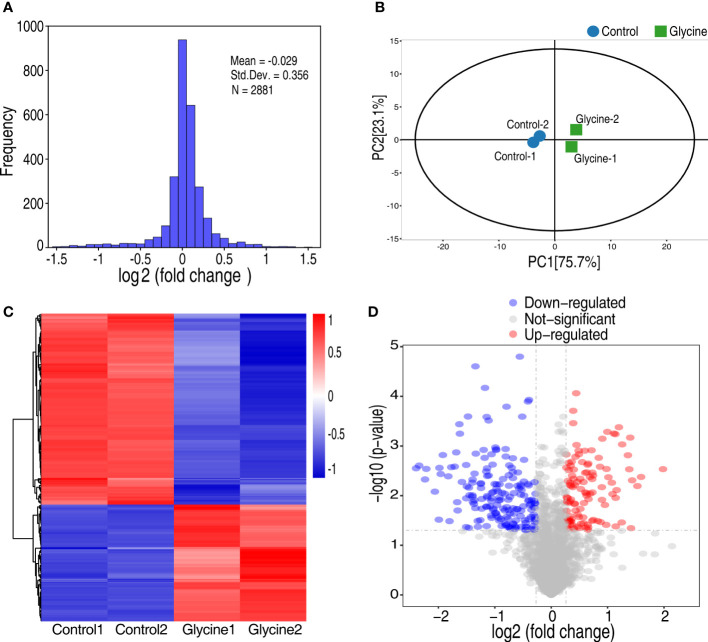
Analysis of glycine on the proteomics of *Vibrio alginolyticus.* **(A)** Frequency distribution of protein ratios (log_2_ scale, glycine vs. control). **(B)** Principal component analysis between glycine and control. Each dot represents the technique replicas in the plot. **(C)** Hierarchical cluster analysis of 291 proteins expressed with statistically significant differences (*p* < 0.05; fold change >1.2 or <0.83) in glycine-treated and untreated (control) samples. **(D)** Volcano plot of the *P*-values vs. the log_2_ protein abundance differences between glycine-treated and control samples. Colors: red, upregulation; blue, down-regulation; gray, non-significant differences.

To better understand how glycine sensitizes *Vibrio* to fish serum, we adopted quantitative proteomics to investigate the glycine-triggered proteomic change. Thus, *V. alginolyticus* 12G01 was treated with glycine for 120 min and then processed for proteomic sample preparation. Proteins were labeled with tandem mass tags (TMT) that are isobaric chemical tags, providing multiplexing capabilities for relative quantitative proteomic analysis (20). The labeled proteins were processed for mass spectrometry analysis. Lastly, a total of 2,881 proteins with unique peptides ≥2 were identified, where the corresponding false discovery rate was less than 0.05%. The ratio of the expression level between the glycine-treated group and the non-treated group was calculated, summarized as frequency, and displayed as histograms. The frequency showed a normal distribution, where the median ratio value was -0.029, implying the protein of altered expression was not skewed (**Figure 2A**). To further demonstrate that glycine induced proteomic change, principal component analysis was adopted, which demonstrated that the control (non-treated bacteria) and the glycine groups (glycine-treated bacteria) were clearly separated from each other (**Figure 2B**). Proteins of differential abundance were screened by setting the abundance ratio at greater than 1.2 or smaller than 0.83, corresponding to increased expression and decreased expression, respectively **Figure 2C**. Meanwhile, the number of matching peptides was greater than or equal to 1. Therefore, 291 proteins of altered abundance were identified, including 111 proteins of increased abundance and 180 proteins of decreased abundance **Figure 2D**. Proteins of differential expression were summarized in **Supplementary Table 1**.

### GO enrichment of differentially expressed proteins upon glycine treatment

**Figure 3:**
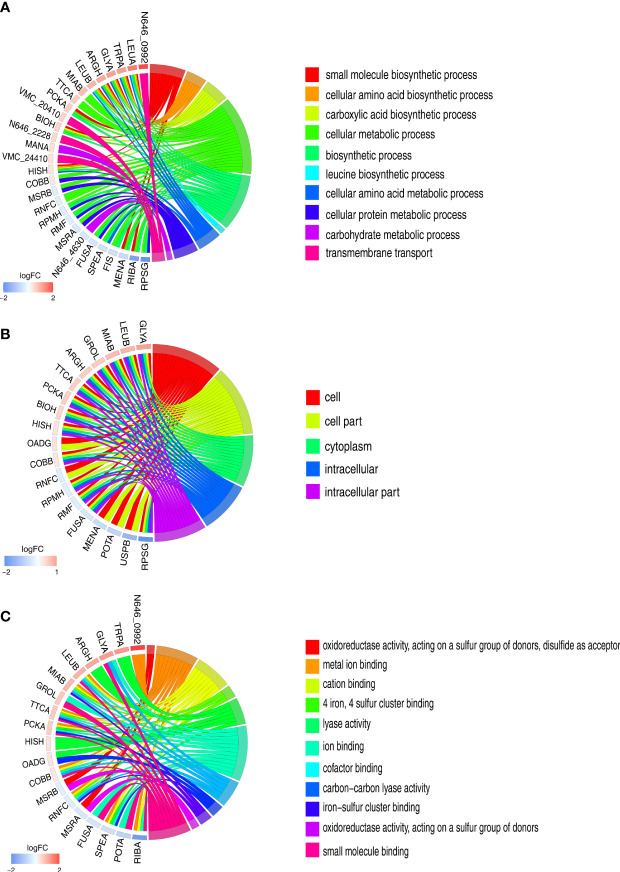
Gene Ontology (GO) term enrichment analysis of differentially expressed proteins. **(A)** Chord chart analysis of GO annotation in biological processes. **(B)** Chord chart analysis of GO annotation in cellular component. **(C)** Chord chart analysis of GO annotation in molecular functions. The left side of the GO Chord displayed whether the gene was up- or down-expression. Red represents up-expression genes and blue represents down-expression. The right side represents different GO terms with different colors. A gene was linked to a certain GO term by the colored bands.

To visualize the parts that were influenced by glycine, the differentially expressed proteins were analyzed with Gene Ontology (GO). The terms of biological process, cellular component, and molecular functions were analyzed. A total of 10 biological processes were enriched **Figure 3A**. Interestingly, all the biological processes were related to metabolism, like biosynthesis of amino acids and carbohydrate metabolism. It is not surprising that glycine triggers a metabolic response since it is itself an amino acid **Figure 3A**. However, it should be noted that glycine affects transport activity as well as that it might have an important function on regulating serum resistance. Consistent with the altered metabolism, differentially expressed proteins were mainly localized in intracellular parts (**Figure 3B**). Moreover, the enrichment of glycine on molecular functions demonstrates that glycine greatly impacts the redox system since four out of 11 molecular functions were oxidoreductase activity or were iron–sulfur proteins (**Figure 3C**).

### Protein–protein interaction network analysis

**Figure 4:**
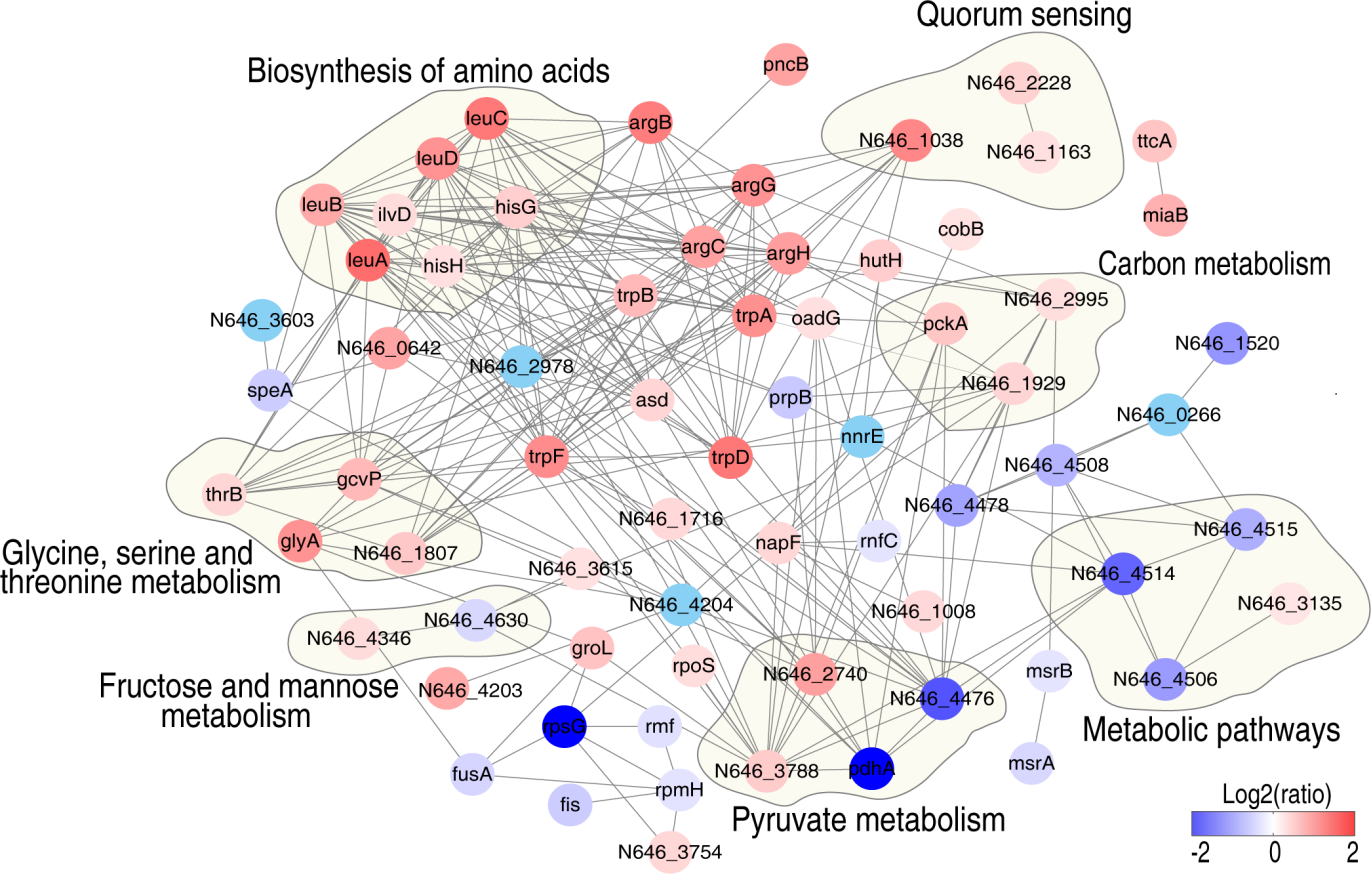
Interaction of differentially expressed proteins in the presence of glycine in *V. alginolyticus*. Protein–protein interaction prediction using STRING software. The circles indicate the enriched Kyoto Encyclopedia of Genes and Genomes pathways. Differently colored dots indicate the altered proteins, blue dots represent downregulated proteins, and red dots represent upregulated proteins, respectively (see the color scale).

An analysis of protein–protein interaction (PPI) may help uncover more proteomic signatures upon glycine treatment (21, 22). Hence, the differentially expressed proteins triggered by glycine were further analyzed by PPI and pathway enrichment analysis (**Figure 4**). Seven pathways were enriched, namely: glycine, serine, and threonine metabolism; fructose and mannose metabolism; pyruvate metabolism; metabolic pathways; carbon metabolism; quorum sensing; and biosynthesis of amino acids. It is not surprising that glycine, serine, and threonine metabolism was enriched since glycine directly activates this metabolic pathway. Four altered proteins (ThrB, GlyA, GcvP, and N646_1807) were identified in the proteomic data. Three proteins were involved in carbon metabolism (PckA, N646_2995, and N646_1929), whose abundance was significantly increased, while two altered proteins related with pyruvate metabolism (PdhA and N646_4476) were largely decreased in terms of abundance. Seven proteins involved in the biosynthesis of amino acids were significantly increased. Interestingly, in addition to glycine, serine, and threonine metabolism, fructose and mannose metabolism connects to pyruvate metabolism and carbon metabolism *via* glycolysis. These data suggest that glycine may impact fructose and mannose metabolism to enhance serum killing.

### Mannitol enhances serum killing of *Vibrios*

**Figure 5:**
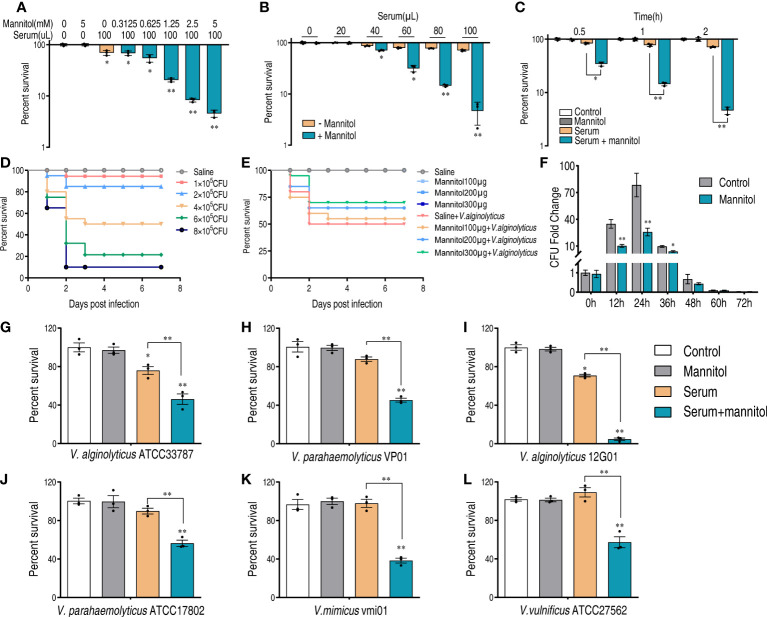
Mannitol promotes the killing effect of serum on *Vibrios*. **(A)** Synergetic effects of 100 μl serum and mannitol on the viability of *V. alginolyticus* 12G01 with the indicated dose of mannitol (0–5 mM). **(B)** Percent survival of *V. alginolyticus* 12G01 incubated with 5 mM mannitol plus serum (0–100 μl) or without mannitol. **(C)** Percent survival of *V. alginolyticus* 12G01 in the presence of 5 mM mannitol or/and 100 μl serum for the indicated length of time. **(D)** Determination of LD50 of *D. rerio* infected with *V. alginolyticus*. Mortality was monitored for 14 days (only 7 days are shown as no death was observed after 7 days). **(E)** Percent survival of *D. rerio* challenged with *V. alginolyticus*, which received mannitol treatment at 1, 4, 10, and 20 h post-infection. Mortality was monitored for 14 days (only 7 days are shown as no death was observed after 7 days). **(F)** The amounts of bacteria in each fish after being treated with or without mannitol (300 μg) and infected with a sublethal dose of bacterial challenge. **(G–L)** Percent survival of *V. alginolyticus* ATCC33787 **(G)**, *V. parahaemolyticus* VP01 **(H)**, *V. alginolyticus* 12G01 **(I)**, *V. parahaemolyticus* ATCC17802 **(J)**, *V. mimicus* vmi01 **(K)**, and *V. vulnificu* ATCC27562 **(L)** in the presence of 5 mM mannitol or/and 100 μl serum. The results are displayed as means ± SEM, and significant differences are identified (*p < 0.05 and **p < 0.01) as determined by Student’s *t*-test. At least three biological repeats were carried out.

In combination with the pathway enrichment analysis and differentially expressed proteins, we speculate that mannitol may be a possible metabolite in enhancing the serum killing activity. There are two reasons for this in the trap-type mannitol/chloroaromatic compound transport system: periplasmic component (encoded by N646_0992) was the most significantly increased protein (3.95 folds, **Supplementary Table 1**), and mannitol could activate fructose and mannose metabolism. Based on this hypothesis, we tested whether mannitol could potentiate serum killing. Mannitol increased the serum killing activity in a dose-dependent manner (**Figure 5A**), and the best efficacy was obtained when mannitol was used at 5 mM. The mannitol-potentiated effect was confirmed by increasing the serum concentration (**Figure 5B**), and mannitol enhanced serum killing in a time-dependent manner (**Figure 5C**).

To confirm this result *in vivo*, we used a *V. alginolyticus*–*Danio rerio* infection model. The half lethal dose (LD50) was determined by infecting *D. rerio* with a series of doses of *V. alginolyticus*. Lastly, 4 × 10^5^ colony-forming units (CFU) were used as LD50 for the following analysis (**Figure 5D**). *D. rerio* was infected with *V. alginolyticus*, followed by administration with saline or different amounts of mannitol as indicated at 1, 4, 10, and 20 h post-infection. Meanwhile, the same amounts of mannitol were injected into *D. rerio* to exclude possible toxic effects. In total, 50% of *D. rerio* without mannitol treatment died, whereas the survival rate was increased in a mannitol dose-dependent manner (**Figure 5E**). When mannitol concentration was used at 300 μg/fish, the best efficacy that increased survival for 20% was observed. Furthermore, the bacterial loads of the whole fish were quantified. The number of bacteria increased at the first 24 h and then decreased afterwards. At 48 h, the number of bacteria was similar to that at 0 h. This was consistent with the death curve in **Figure 5E** in that *D. rerio* mainly died at the first 48 h. It is noticeable that the presence of mannitol decreased the bacterial loads from 12 to 48 h and that these were the same regardless of the application of the mannitol treatment or not (**Figure 5F**). Furthermore, mannitol exerted a serum-promoting effect to other *Vibrio* strains including *V. alginolyticus* ATCC33787, *V. parahaemolyticus* VP01, *V. alginolyticus* 12G01, *V. parahaemolyticus* ATCC17802, *V. mimicus* vmi01, and *V. vulnificus* ATCC27562 (**Figures 5G–L**). These data suggest that mannitol promotes serum killing *in vitro* and bacterial clearance *in vivo*.

### Mannitol increases the activity of glycolysis and the pyruvate cycle

**Figure 6:**
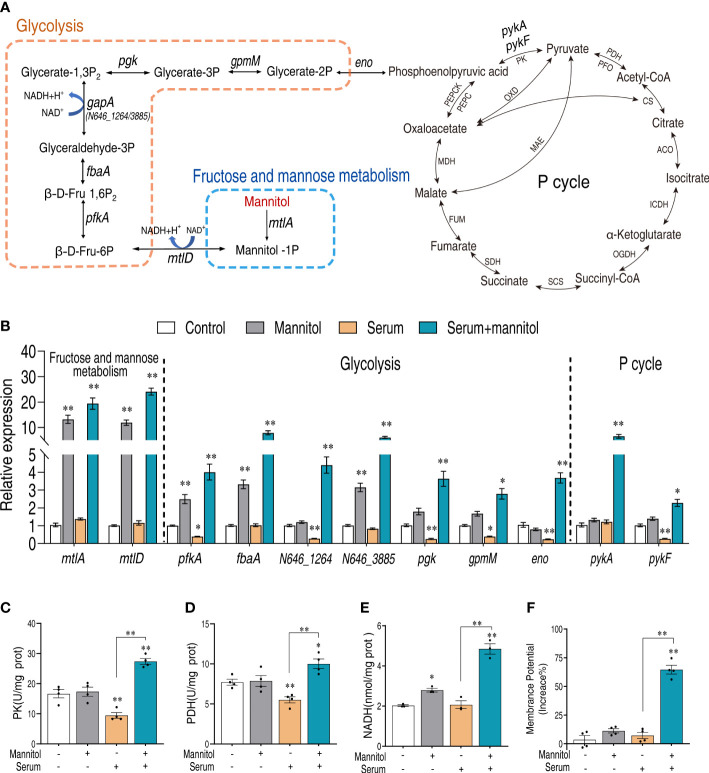
Mannitol promotes glycolysis and the pyruvate cycle. **(A)** Mannitol metabolism pathway. **(B)** Real-time quantitative reverse transcription-PCR (qRT-PCR) for the quantification of gene expression of glycolysis and P cycle. **(C, D)** Activity of pyruvate kinase and pyruvate dehydrogenase in the presence of mannitol or/and serum. **(E)** Effects of mannitol or/and serum on the content of NADH. **(F)** Membrane potential of *Vibrio alginolyticus* treated with 100 μl serum for 2 h in the presence or absence of 5 mM mannitol. The results are displayed as means ± SEM, and significant differences are identified (*p < 0.05 and **p < 0.01) as determined by Student’s *t*-test. At least three biological repeats were carried out.

There are two possibilities that mannitol may enhance the serum killing activity. One is that mannitol directly regulates the serum killing activity, and the other one is that the mannitol metabolism contributes to the serum killing activity. We speculate that the latter is the possibility since mannitol can be phosphorylated to form mannitol-1P by D-mannitol PTS permease during the transport (23, 24) (**Figure 6A**). To test this possibility, the expression levels of mannitol metabolism were quantified in the control, mannitol only, serum only, and mannitol plus serum groups. Mannitol alone or mannitol plus serum significantly increased the gene expression of glycolysis, especially for *pfkA* that was repressed by serum (**Figure 6B**). More importantly, several of the genes (N646_1264, *pgk*, *gpmM*, and *eno*) were decreased by the presence of serum but can be rescued by mannitol, suggesting that serum impaired glycolysis. Glycolysis is upstream of the pyruvate cycle (25–28). Similarly, the expression of the genes (*pykA* and *pykF*) that connect glycolysis and the pyruvate cycle was decreased by serum but enhanced by glycine plus serum.

To further confirm this result, the enzymatic activities of the pyruvate cycle, pyruvate kinase (PK), and pyruvate dehydrogenase (PDH) were investigated. Serum consistently decreased the activities of the two enzymes, but the presence of mannitol increased their activity (**Figures 6C, D**). The activated glycolysis and pyruvate cycle, by the presence of mannitol and serum, were confirmed with the content of NADH (**Figure 6E**) and proton motive force (**Figure 6F**). These data together suggest that mannitol enhances glycolysis and the pyruvate cycle that enhance serum killing.

### Mannitol enhances complement deposition *via* glycolysis and the pyruvate cycle

**Figure 7:**
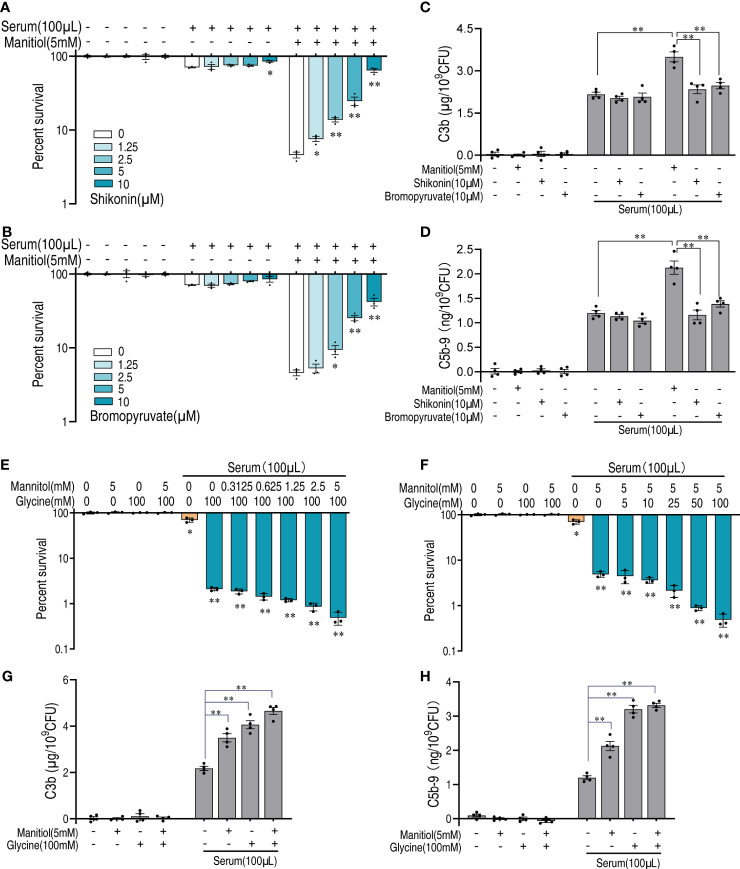
Mannitol increases serum sensitivity *via* glycolysis and the pyruvate cycle. **(A, B)** Percent survival of *V. alginolyticus* in the presence or absence of increasing doses of shikonin **(A)** or bromopyruvate **(B)** and in the presence of serum and/or mannitol. **(C, D)** Quantification of C3b **(C)** and C5b-9 **(D)** incubated with 10 μM shikonin or 10 μM bromopyruvate in the presence or absence of 100 μl serum alone or in the presence of or without mannitol (5 mM). **(E)** Percent survival of *V. alginolyticus* 12G01 incubated with 100 mM glycine plus serum (100 μl) in a mannitol dose-dependent manner (0–5 mM). **(F)** Percent survival of *V. alginolyticus* 12G01 incubated with 5 mM mannitol plus serum (100 μl) in a glycine dose-dependent manner (0–100 mM). **(G, H)** Quantification of C3b **(G)** and C5b-9 **(H)** incubated with 5 mM mannitol and/or 100 mM glycine in the presence or absence of 100 μl serum. The results are displayed as means ± SEM, and significant differences are identified (*p < 0.05 and **p < 0.01) as determined by Student’s *t*-test. At least three biological repeats were carried out.

As suggested by the abovementioned results that glycolysis is critical for the function of mannitol, we treated bacteria with inhibitors shikonin and bromopyruvate that target pyruvate kinase and pyruvate dehydrogenase, respectively. Either shikonin or bromopyruvate alone had a limited effect on bacterial survival. The killing of bacteria by mannitol and serum was blocked by either treatment and showed a dose-dependent manner (**Figures 7A, B**). The deposition of complement to the bacterial surface is believed to be the key part in enhancing serum lytic activity. Mannitol increased the amount of complements C3b and C5b-9 on the bacterial surface (**Figures 7C, D**). However, shikonin or bromopyruvate decreased the amount of deposited complement components. Moreover, we tested the synergistic effects of glycine and mannitol on serum killing. Mannitol had a limited effect on glycine-enabled serum killing (**Figure 7E**), which was the same as the effect of glycine on mannitol (**Figure 7F**). Consistently, mannitol and glycine do not mutually promote each other’s ability on C3b and C5b-9 deposition (**Figures 7G, H**). Thus, these data suggest that mannitol is downstream of glycine whose action is dependent on glycolysis and the pyruvate cycle to enhance complement deposition to promote killing.

## Discussion

Managing a bacterial infection is critical for sustainable agriculture and human health. Antibiotics are the major choice for the control of a bacterial infection. However, this option is challenged in recent years because of the spread of antibiotic-resistant bacteria (29). Policies have been issued to ban the use of antibiotics as feed additive in agriculture, which presents a severe threat for the production of food animals, in many countries (30, 31). Therefore, alternatives to control a bacterial infection is urgently needed.

Harness host’s immune system has been a hot spot for identifying potential targets for the control of a bacterial infection. The complement system is a key part in defending against bacterial pathogens. However, serum resistance is a common virulent trait for different pathogens (32, 33). Therefore, reversing serum resistance is a potential way to reduce bacterial loads to a level that can be eliminated by other parts of the immune system. One challenge for such study is the diversified mechanisms utilized by bacterial pathogens to escape serum killing. Different bacteria use different virulent factors to disrupt the normal function of the complement system (34). This is the possible reason that there is still lack of proper control measures. This challenge is addressed by a recent report stating that metabolic reprogramming of serum-resistant bacteria can be a potential way to enable serum killing. Glycine was identified to be a metabolite to reverse serum resistance, but its efficacy varied from pathogen to pathogen. Thus, identifying the underlying mechanism would be a way to find other metabolites that can reverse in a different pathogen.

Deposition of complement components to the bacterial surface is crucial for the action of a complement system that either directly lyse bacteria *via* membrane-attached complex or opsonized bacteria toward immune cells for phagocytosis or triggering an adaptive immune response (35). As such, promoting complement deposition is a strategy to overcome complement resistance. In this study, we quantify how mannitol promotes C3b and C5b-9 deposition, which are the terminal complex for complement activity. C3 is located at the hub where all three complement pathways—classic pathway, lectin pathway, and alternative pathway—converge and activate C3 by proteolytic cleavage that form C3b. C3b covalently attaches to the bacterial surface for the following immune activation. C3b also activates C5 by splitting which generates C5b to form membrane-attached complexes with C6, C7, C8, and C9 at the bacterial surface (36). Thus, the deficiency of C3 leads to severe a bacterial infection, including those caused by meningococci and pneumococci (37). The exogenous administration of complement component C3 and the active form C3a in Japanese flounder increased its survival against bacterial infection by inducing chemotaxis to peripheral blood leukocytes (38). Given the importance of C3 and C5b-9, enhancing C3 and C5-9 deposition is key to boost the complement killing activity.

Taking advantage of proteomics, we find that the expression of transporters for mannitol are increased, which motivates us to investigate the potential function of mannitol in reversing serum resistance. Interestingly, mannitol promotes serum killing effects, and we find that this effect is dependent on glycolysis and the pyruvate cycle (25, 26, 28). We have previously shown that the pyruvate cycle regulates the TCA cycle in *Escherichia coli*, *Edwardsiella tarda*, and *V. alginolyticus* (28, 39). Thus, *V. alginolyticus* contained full sets of glycolytic genes and pyruvate cycle genes. Both of glycolysis and the pyruvate cycle are energy metabolism that provides energy for their growth and virulence. Glycolysis is critical for *Vibrio* growth as they predicated highly expressed genes to support their fast growth (40). Modulation of such energy metabolism is crucial for the bactericidal activity disinfectant. Slightly acidic electrolyzed water, acidic electrolyzed water, and sodium hypochlorite, commonly used to inactivate the food-born pathogen *V. parahaemolyticus*, decreased the expression of adenylate kinase, phosphoglycerate kinase, glyceraldehyde-3-phosphate dehydrogenase, and enolase to exert an antibacterial activity (41). Moreover, central carbon metabolism is strongly associated with antibiotic resistance in *Vibrio*. Decreased glycolytic activity and pyruvate cycle activity is associated with levofloxacin resistance, gentamycin resistance, and colistin resistance (25, 42). The mechanism is that the downregulation of glycolysis and the pyruvate cycle reduced the intracellular generation of reactive oxygen species, whereas reactivating glycolysis and the pyruvate cycle, *e*.*g*., glucose, re-sensitizes antibiotic-resistant bacteria to antibiotics (25, 43). Recent studies also show that the glycolytic pathway and the pyruvate cycle are subjected to the regulation of acetylation and succinylation, indicating that protein post-translation modification may be involved in serum resistance (44–46). Actually, our previous study demonstrated that the activity of the pyruvate cycle was decreased by serum (17). Here we complement this finding that it is the serum that decreased the glycolytic pathway to repress glycolysis by withdrawing fuel sources.

Although there is no direct evidence to demonstrate how serum modulates the bacterial physiology, we postulate two possibilities. First, serum contains certain metabolite/factors that can be utilized by bacteria to remodel their metabolism to prevent complement killing—for example, the recent proposal of microbial endocrinology suggests that signals of the host endocrine system impact the microbial physiology. Epinephrine, norepinephrine, dopamine, estriol, estradiol, estrogen, and progesterone influence bacterial growth, bacterial resistance to antimicrobial peptides, quorum sensing, biofilm formation, and virulent gene expression such as *E. coli* adhering to the intestinal epithelial cells in pigs (47–56). It is highly possible that such signal may participate in serum resistance, which requires further investigation. Second, serum induces a stress response that causes a global metabolomic shift. Our previous study showed that glycine downregulates cAMP/CRP transcriptional factors. Since this transcription factor is involved in both metabolism and stress response, it is not surprising that bacteria activate cAMP/CRP to defend against serum killing but decrease metabolism as well. Thus, it makes sense that enhancing metabolism by glycine or mannitol reverses this phenotype.

In summary, we demonstrate that mannitol is a downstream metabolite effector induced by glycine. Mannitol flux into glycolysis to drive the activation of the pyruvate cycle, which is critical to enhance the serum killing activity. This effect was confirmed both *in vitro* and *in vivo*. Our study paves a possible way to develop an eco-friendly compound in aquaculture to treat bacterial infections.

## Materials and methods

### Ethics statement

All animal experiments were reviewed and approved by the Institutional Animal Care and Use Committee of Sun Yat-sen University (approval no. SYSU-IACUC-2020-B126716).

### Bacterial strain

Bacterial strains *V. alginolyticus* ATCC33787, *V. parahaemolyticus* ATCC17802, and *V. vulnificus* ATCC27562 and clinical isolates including *Vibrio alginolyticus* 12G01, *V. parahaemolyticus* VP01, and *V. mimicus* vmi01 (42, 57) were among the collection of our laboratory. Bacteria were grown in standard Luria–Bertani broth medium (1% bacterial peptone and 0.5% yeast extract) plus 3% NaCl and were propagated at 30°C. The bacterial culture grown overnight was diluted to 1:100 using fresh medium and grown until the optical density was 0.5. Bacterial cells were collected by centrifugation and were washed three times with saline solution.

### Fish and rearing conditions

Nile tilapia (*Oreochromis niloticus*) with an average weight of 500 ± 10 g was obtained from a local fish company (Guangdong Tilapia Breeding Farm, Guangzhou, China). The tilapia was maintained in 180-L tanks whose parameters were set as follows: water temperature at 28°C, pH value of 7.0–7.5, carbon dioxide <10 mg/L, oxygen content of 6–7 mg/L, and nitrogen content of 1–2 mg/L. The tilapia was acclimated for at least 2 weeks before the experimental manipulation (58–60).

Zebrafish with an average body weight as 0.22 ± 0.03g was obtained from a local zebrafish corporation (Guangzhou, China). Before the experiment, the fish was tested by microbiological detection to ensure that it is free from *Vibrio* species infection. The zebrafish was cultured in a 25-L open-circuit filtered water tanks at room temperature with aeration (39, 61, 62). The animals were maintained in the condition stated above for 2 weeks before the experiment. The zebrafish was fed twice per day with commercial blood worm.

### Protein sample preparation and TMT labeling

Bacterial colonies were inoculated in a 5-ml medium and cultured at 30°C. The overnight cultures were re-inoculated into 100 ml medium with 1:100 ratio, followed by growth until OD600 was 0.5. Bacterial cells were collected by centrifugation, washed three times with saline solution, and resuspended until OD600 was 1.0. An aliquot of 3 ml bacteria was collected in a 5-ml centrifugation tube, and glycine dissolved in saline solution was added to the bacterial solution to a final concentration of 100 mM. Bacteria treated with saline solution only was established as the control group. Afterwards, after 2 h of incubation, bacteria were collected for protein labeling. For each sample, two biological replicates were performed. The collected cells were lysed with lysis buffer. Trypsin (sequencing grade, Promega, Madison, WI, USA) was used to digest the proteins. Then, the generated peptide mixture was labeled as constructed by TMT kit (Thermo Fisher Scientific, USA). The labeled peptides were desalted with C_18_ desalting column (Sigma Aldrich, USA). The polypeptide sample of 100 μg was separated by reverse-phase ultra-performance liquid chromatography under alkaline conditions and then identified using mass spectrometry (63).

### Proteomics analysis

For each fraction, 1 μg peptide was separated with a nano-UPLC (EASYnLC1200) coupled to a Q Exactive HFX Orbitrap instrument (Thermo Fisher Scientific) that is equipped with a nano-electrospray ion source. The peptide mixture was separated by a reverse-phase column (100 μm ID × 15 cm, ReprosilPur120 C_18_AQ, 1.9 μm, Dr. Math). The mobile phases contained 0.1% FA and 2% ACN (phase A) as well as 90% CAN and 0.1% FA (phase B). Separation was carried out for a total of 90 min, with a flow rate of 300 nl/min. Gradient B was performed as follows: 2% to 5% for 2 min, 5% to 22% for 68 min, 22% to 45% for 16 min, 45% to 95% for 2 min, and 95% for 2 min. Raw MS files were exported and analyzed with Max Quant software (version 1.6.15.0). Carbamidomethyl [C] was set as a fixed modification, while oxidation (M), acetyl (protein N term), deamidation (N), and deamidation (NQ) were set as variable modifications (the maximum number of variable modifications was five per peptide). The false discovery rate was set to 0.01 for both protein and peptide, where the minimum length for a peptide was seven amino acids. Proteins of altered expression were selected if their folds of change <0.83 or >1.2) (64) and with a *P*-value ≤0.05 (65, 66).

### Sample preparation and serum killing

Fish serum was collected from 50 fish with equal numbers of male and female individuals. Serum was collected *via* a vein puncture and was isolated by centrifugation at 3,000 × g for 10 min at 4°C. The sera were aliquoted to a small volume and stored at −80°C (17). The sera were used only one time to avoid the thaw–frozen cycle. To perform serum killing, bacteria were resuspended in saline solution to OD600 of 1.0. Then, 3 ml of the bacteria was collected by centrifugation and resuspended in 100 μl serum or/and metabolites. The bacteria were incubated at 30°C for 2 h with shaking. After that, bacteria were collected to determine colony-forming units by plating.

### Quantitative real-time PCR

qRT-PCR was performed as previously described (67, 68). Total RNA was extracted from *V. alginolyticus* with TRIzol reagent (Invitrogen, USA). The quality and the purity of RNA were checked by electrophoresis and Nanodrop. Reverse transcription was performed with EvoM-MLV RT kit with gDNA clean for qPCR (AG11705; Accurate Biology). The reaction for qRT-PCR was conducted in a 10-μl reaction including 5 μl 2× SYBR green premix pro Taq HS qPCR kit (AG11701; Accurate Biology), 2.6 μl H_2_O, 2 μl cDNA template, and 0.2 μl forward and reverse primers. Three technical replicas were included for each sample, and the reaction was run on a CFX384 Touch (Bio-Rad, USA).

### Bacterial infection

Overnight bacteria were diluted 1:100 in growth medium until OD600 was 1.0 at 30°C (69–71). Bacteria were collected for bacterial challenge. The zebrafish were randomly grouped and injected intramuscularly with 5 μl of 1 × 10^5^ CFU, 2 × 10^5^ CFU, 4 × 10^5^ CFU, 6 × 10^5^ CFU, or 8 × 10^5^ CFU bacteria or saline solution (*n* = 30 for each treatment). Death of fish was checked twice a day for a total of 14 days to obtain the accumulative death.

### Measurement of NAD^+^/NADH

NAD^+^/NADH measurement was performed as previously reported (26, 72, 73). Bacteria were suspended in saline solution to 1.0 of OD600. Furthermore, 2 ml of bacteria was resuspended in NADH/NAD^+^ extraction buffer and incubated in 60°C water for 5 min, and then the assay buffer was added. After vertexing and centrifugation, the supernatant was collected and read according to the protocol (EnzyChrom NAD^+^/NADH Assay Kit, BioAssay Systems).

### Measurement of membrane potential

Membrane potential was measured as reported (73, 74). Briefly, the bacteria (1 × 10^6^ CFU) were stained with 3,3′-diethyloxacarbocyanine iodide for 30 min. The reading was performed by flow cytometry (FACSCalibur flow cytometer, Becton Dickinson, San Jose, CA, USA).

### Measurement of enzyme activity

The activity of PDH was measured as reported (75–77), and the PK enzymatic activity was assessed using a commercial assay kit (BC0545, Beijing Solarbio Science and Technology Co., Ltd.). The bacteria were treated as stated above. After incubation with serum or/and mannitol, the bacteria were collected and adjusted to OD600 of 1.0. Moreover, 20 ml of OD600 = 1.0 bacterial solution was collected in a 1.5-ml centrifugation tube, resuspended with 1 ml sterile saline, and disrupted by sonic oscillation (200 W total power with 35% output, 2 s pulse, and 3 s pause over ice) for 3 min. After centrifugation, the supernatants of the enzyme activity assay were collected. Protein concentration was measured with enhanced BCA Protein Assay Kit (P0010, Beyotime, Shanghai, China).

### Quantification of C3b/C5b-9 deposited on the bacterial outer membrane

Commercial assay kits (FS-E63210-96T and A119939-96T, Shanghai Fusheng Industrial Co., Ltd.), were used to quantify the C3b/C5b-9 on the bacterial outer membrane. The bacteria were treated as described above and were processed for ELISA.

## Data availability statement

The data presented in the study are deposited in ProteomeXchange Consortium via the iProX partner repository(78), accession number PXD035517.

## Ethics statement

The animal study was reviewed and approved by Institutional Animal Care and Use Committee of Sun Yat-sen University.

## Author contributions

T-sK and J-hW conducted the experiments. T-sK, J-hW, and X-wC performed data analysis. T-sK, X-wC, and BP interpreted the data. BP wrote the manuscript. BP conceptualized and designed the project. All the authors reviewed the manuscript and acknowledged the contributions. All authors contributed to the article and approved the submitted version.

## Funding

This work was sponsored by grants from the National Natural Science Foundation of China (NSFC) project [32061133007, 31872602], Project supported by Innovation Group Project of Southern Marine Science and Engineering Guangdong Laboratory (Zhuhai) [No. 311020006], and The Youth Talent Support Program of Guangdong Province [2017GC010617] (to B. P.).

## Acknowledgments

We also thank Dr. D.X. Yang and Mr. Y.C. Cao for their assistance in the collection of fish serum.

## Conflict of interest

The authors declare that the research was conducted in the absence of any commercial or financial relationships that could be construed as a potential conflict of interest.

## Publisher’s note

All claims expressed in this article are solely those of the authors and do not necessarily represent those of their affiliated organizations, or those of the publisher, the editors and the reviewers. Any product that may be evaluated in this article, or claim that may be made by its manufacturer, is not guaranteed or endorsed by the publisher.
